# More than ten years without changes in the prevalence of adverse food reactions among Mexican adults: Comparison of two cross-sectional surveys

**DOI:** 10.5415/apallergy.0000000000000163

**Published:** 2025-09-10

**Authors:** Jaime Morales-Romero, Misael Aguilar-Panduro, Tonatiuh Ramses Bedolla-Pulido, Dante Daniel Hernández-Colín, María Enriqueta Nuñez-Nuñez, Martín Bedolla-Barajas

**Affiliations:** 1 Universidad Veracruzana, Instituto de Salud Pública, Xalapa, Veracruz, México; 2 Universidad de Guadalajara, Centro Universitario del Sur, Escuela de Medicina, Ciudad Guzman, Jalisco, México; 3 Departemento de Alergia e Inmunología Clínica, Nuevo Hospital Civil de Guadalajara “Dr. Juan I. Menchaca,” Guadalajara, Jalisco, México

**Keywords:** Adult, cross-sectional study, food hypersensitivity, prevalence, risk factors, trend

## Abstract

**Background:**

Some regions of the world have experienced increases in the prevalence of diseases such as asthma, allergic rhinitis, and atopic dermatitis; however, little is known about whether the same has occurred with the prevalence of adverse food reactions.

**Objective:**

To determine the temporal trend in the prevalence of adverse food reactions among adults in western Mexico.

**Methods:**

The results of 2 cross-sectional studies conducted 11 years apart (2012–2013 and 2023-2024) are compared. Data were obtained through a standardized survey administered to adults participating in physical activities in the “vía recreactiva” of Guadalajara and were included through stratified sampling based on sex and age.

**Results:**

The first and second studies included 471 and 485 adults, respectively. Comparatively, the prevalence of adverse food reactions was 19.5% and 19.4% (*P* = 0.953). For oral allergy syndrome, urticaria, and self-reported anaphylaxis, the prevalence was 6.8% and 9.7% (*P* = 0.104), 5.9% and 4.3% (*P* = 0.258), and 2.3% and 0.6% (*P* = 0.032), respectively. Multivariate analyses identified female sex (adjusted odds ratio: 1.52, *P* = 0.013), age between 25 and 50 years (adjusted odds ratio: 0.59, *P* = 0.002), personal history of allergic rhinitis (adjusted odds ratio: 2.10, *P* = 0.004), and atopic dermatitis (adjusted odds ratio: 4.99, *P* < 0.001) as factors associated with adverse food reaction.

**Conclusion:**

Overall, the prevalence of adverse food reactions, their clinical manifestations, and the most implicated foods did not change during the analysis period. Sex, age, and atopic comorbidities were associated with adverse food reactions.

## 1. Introduction

The prevalence of adverse food reactions (AFR) in adults has been estimated in various studies worldwide, with figures ranging from 9.3% to 32% in the adult population [1–4].

Food allergies have emerged as a significant global health issue in recent decades, with notable changes in their prevalence worldwide. In the Americas, countries such as the United States and Canada have seen upward changes, with increases of 44% and 32% over a period of 10 and 6 years, respectively [3, 5]. In Europe, the increase was nearly 20% over a 10-year period [4]; meanwhile, in Asia, many more significant changes have been observed, with a 95% increase over a period of 13 years [6]. These changes reflect a global upward trend in food allergy, highlighting the need for a better understanding of this issue.

Paradoxically, in Latin America, no studies have been conducted to assess whether the prevalence of food allergies has followed the same global trend, which becomes more urgent when considering that most studies conducted to evaluate this phenomenon have primarily focused on the pediatric population. With this in mind, the aim of our study was to determine the temporal trend in the prevalence of AFR in adults in western Mexico, whether the symptoms and foods associated with this condition have experienced changes, and what factors are associated with AFR.

## 2. Methods

### 2.1. Ethics

The study protocol was approved by the Ethics and Research Committee of the Civil Hospital of Guadalajara Dr. Juan I. Menchaca (Number CEI-00087); Chairperson Aguilar J, MD) on November 10, 2023, and the Municipal Sports Council. Our study adhered to the principles of the Declaration of Helsinki regarding research involving human subjects. Verbal approval to answer the survey was considered consent to participate in the study. The confidentiality of participants was protected by conducting the survey anonymously.

### 2.2. Setting

The present study retrieved data from a previous study [7] for comparison with those obtained in a new survey. In the previous study, most participants were residents of Guadalajara, which was chosen as the location for this new research. Guadalajara, located in the western region of Mexico and characterized by a temperate subhumid climate, is recognized as the second most populous city in the country, with approximately 1,385,629 inhabitants according to 2020 data provided by the Institute of Statistical and Geographic Information [8].

### 2.3. Design

Over a period of 11 years, 2 cross-sectional surveys of the same study population were conducted: the first from December 2012 to April 2013 and the second from December 2023 to February 2024. This approach yielded 2 independent samples: the first from the 2012 to 2013 cross-sectional survey and the second from the 2023 to 2024 cross-sectional survey. Both samples included young adults (18–24 years) and adults (25–50 years) who visited the “vía recreactiva” of Guadalajara every Sunday to engage in physical activity. Four trained interviewers carried out probabilistic sampling stratified by age and sex. In both cross-sections (2012–2013 and 2023–2024), 4 strata were defined as follows: (1) men from 18 to 25 years old, (2) women from 18 to 25 years old, (3) men from 26 to 50 years old, and (4) women from 26 to 50 years old. The sample size of the population analyzed from 2012 to 2013 (n = 471) was decided by proportional allocation of a larger sample, as explained in the methodology of a previous study [7]. The criteria were an expected prevalence of AFR of 12%, a maximum allowable error of 3%, and a confidence level of 99.5%. The size of the resulting sample was adjusted proportionally to the population of the city of Guadalajara, which represents 42% of the metropolitan area to which it belongs. The final sample size was n = 471. In the second analysis of 2023–2024, a sample size similar (n = 485) to that of the previous study was defined. After verifying the selection criteria, the subjects were selected as they were identified consecutively, according to the stratum to which they belonged, until the sample size was complete.

### 2.4. Instrument

Data were obtained using a structured questionnaire. Participants were consecutively included in the study. After obtaining consent to participate, they were asked if they lived in the city of Guadalajara. If they answered affirmatively, a questionnaire was administered. The questionnaire included questions about age, sex, and symptoms after consuming food and triggering foods. It also inquired about the personal history of allergic diseases, such as asthma, allergic rhinitis (AR), or atopic dermatitis (AD) (Supplementary Material 1, http://links.lww.com/PA9/A43).

### 2.5. Definitions

AFR was defined as an affirmative response to the question “Do you suffer from allergic reactions after eating or drinking?”

A convincing reaction of nonsevere and severe food allergies was defined according to Gupta et al. [9]. The first refers to cases where the subject reports a stringent symptom with involvement of a single organ (eg, urticaria, angioedema, pharyngeal or chest tightness, wheezing, vomiting, among others). The second refers to the presence of stringent symptoms with the involvement of two or more organs. The remaining cases were considered as food intolerance.

Oral allergy syndrome is defined as the presence of symptoms in the oral cavity. Urticaria was defined as the presence of hives accompanied by body itching, both occurring after food consumption. To identify potential cases of food-induced anaphylaxis, participants were asked if they were aware of anaphylaxis due to food consumption (self-reported anaphylaxis).

### 2.6. Analysis

For each study, the prevalence of AFR was determined by dividing the number of subjects who answered affirmatively to the question “Do you suffer from allergic reactions after eating or drinking?” on the basis of the total number of participants. A similar procedure was done for a convincing nonsevere and severe food allergies (FA).

Qualitative variables were expressed as frequencies and proportions were compared using Pearson chi-square test. To identify trends in the prevalence of AFR, a convincing nonsevere and severe FA, and its symptoms over an 11-year period, the chi-square test was used.

Multivariate logistic regression analysis was conducted to identify factors associated with AFR (dependent variable). The predictor variables introduced into the model were sex, age, and personal history of asthma, AR, and AD. Statistical significance was set at *P* ≤ 0.05. Data analyses were performed using IBM SPSS Statistics 29 (IBM Corp., Armonk, NY, USA).

## 3. Results

### 3.1. Description of the populations

The 2012–2013 cross-sectional sample consisted of 471 adults (49.5% women; mean age, 28.9 years), while the 2023–2024 cross-sectional sample included 485 adults (50.5% women; mean age, 27.3 years). When comparing both groups, no significant differences were observed in terms of age or sex distribution (*P* > 0.05). Similarly, the prevalence of asthma, AR, AD, urticaria, and drug allergies was similar between the 2012 to 2013 and 2023 to 2024 cross-sectional samples (*P* > 0.05) (Table 1).

**Table 1.** Demographic and clinical characteristics of the study populations

	2012–2013	2023–2024	
	n = 471	n = 485	*P*
Age, y			0.700
18–24	237 (50.3)	238 (49.1)	
25–50	234 (49.7)	247 (50.9)	
Sex			0.746
Male	238 (50.5)	240 (49.5)	
Female	233 (49.5)	245 (50.5)	
Personal atopy			
Asthma	30 (6.4)	34 (7.0)	0.692
Allergic rhinitis	36 (7.6)	55 (11.3)	0.052
Atopic dermatitis	21 (4.5)	23 (4.7)	0.834
Urticaria	16 (3.4)	15 (3.1)	0.791
Drug allergy	50 (10.6)	40 (8.2)	0.210

In parentheses: proportions. n: subjects selected according to the cross-sectional period. Comparison of proportions using the chi-square test.

### 3.2. Prevalences of AFR symptoms

**Figure 1.**
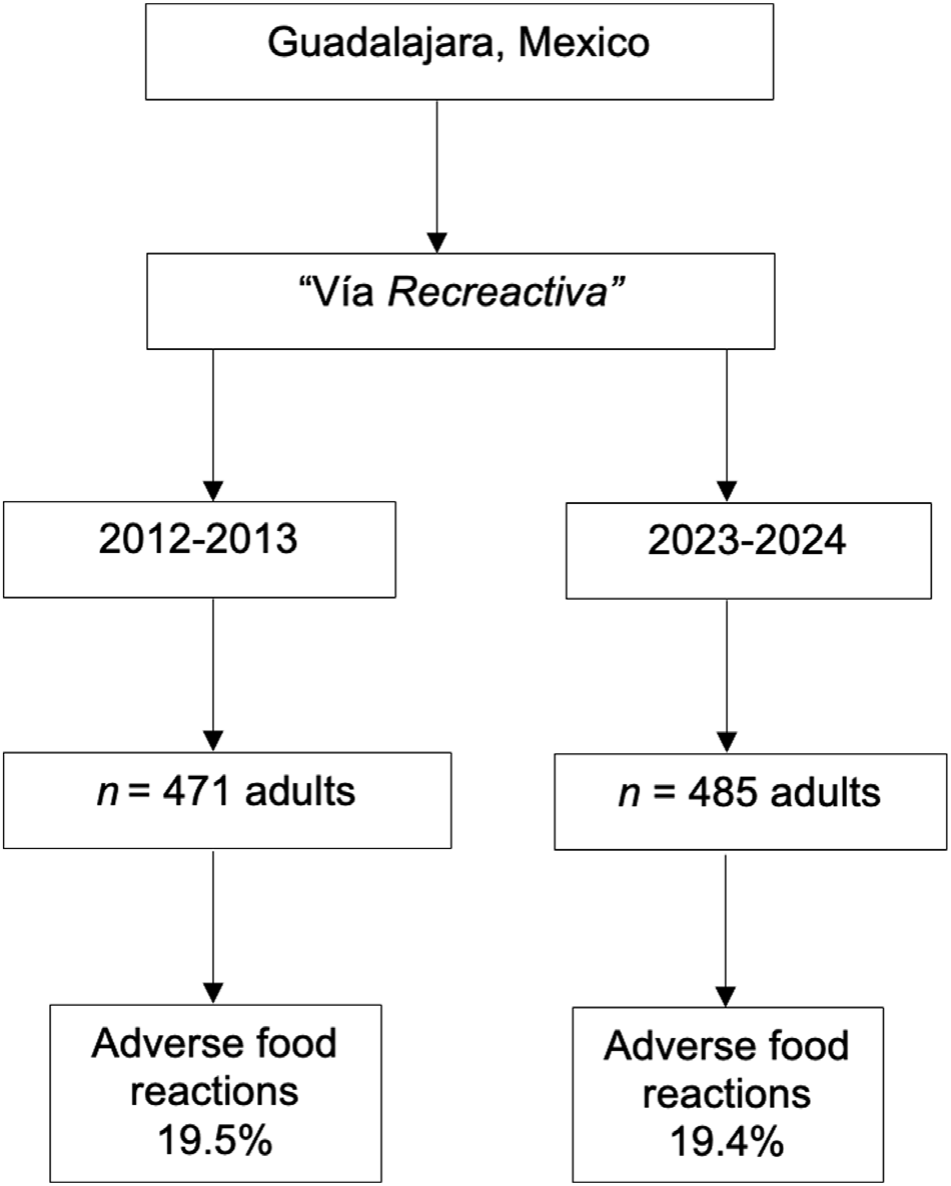
Flow diagram of the selection of study subjects and the comparison of the prevalence of adverse food reactions in 2 sections in time

Notably, the prevalence of AFR did not differ significantly between the 2 study periods (19.5% vs 19.4%, *P* = 0.953) (Fig. 1).

The prevalence of convincing food allergy, nonsevere convincing food allergy, and severe convincing food allergy in the study periods was 8.5% (40/471) and 6.2% (30/485) (*P* = 0.171), 8.3% (39/471) and 5.6% (27/485) (*P* = 0.10), and 0.2% (1/471) and 0.6% (3/485) (*P* = 0.65), respectively. The prevalence of food intolerance was 11.0% (52/471) in 2012 to 2013 and 13.2% (64/485) in 2023 to 2024 (*P* = 0.31).

In cases of oral allergy syndrome and urticaria, the prevalence was similar: 6.8% and 9.7% (*P* = 0.104) and 5.9% and 4.3% (*P* = 0.258), respectively. However, self-reported anaphylaxis showed a downward trend, decreasing from 2.3% to 0.6% (*P* = 0.027). Regarding nonspecific symptoms, such as headache, vomiting, abdominal pain, or flatulence, only abdominal distension was significantly higher in the 2023 to 2024 cross-sectional sample (1.5% vs 3.5%, *P* = 0.046) (Table 2).

**Table 2.** Frequency of adverse food reactions symptoms among adults

	2012–2013	2023–2024	
	n = 471	n = 485	*P*
Adverse food reaction	92 (19.5)	94 (19.4)	0.953
Oral allergy syndrome	32 (6.8)	47 (9.7)	0.104
Urticaria	28 (5.9)	21 (4.3)	0.258
Self-reported anaphylaxis	11 (2.3)	3 (0.6)	0.027
Headache	13 (2.8)	9 (1.9)	0.351
Vomiting	13 (2.8)	8 (1.6)	0.241
Abdominal pain	9 (1.9)	17 (3.5)	0.130
Abdominal distension	7 (1.5)	17 (3.5)	0.046
Diarrhea	6 (1.3)	13 (2.7)	0.119
Flatulencies	3 (0.6)	5 (1.0)	0.726
Chest pain	0 (0)	5 (1.0)	0.062

In parentheses: proportions. n: subjects selected according to the cross-sectional period. Comparison of proportions using the chi-square test for trend.

### 3.3. Prevalence of AFR by sex and age

When comparing the prevalence of AFR by sex between the 2012 to 2013 and 2023 to 2024 cross-sectional samples, it was observed that the prevalence in women and men was 21.9% and 24.5% (*P* = 0.501) and 17.2% and 14.2% (*P* = 0.358), respectively. When the prevalence of AFR was estimated according to age group, it was 25.7% vs 21.0% (*P* = 0.223) in the 18 to 24 year age group and 13.2% vs 17.8% (*P* = 0.168) in the 25 to 50 year age group.

### 3.4. Foods associated with AFR and convincing FA

**Figure 2.**
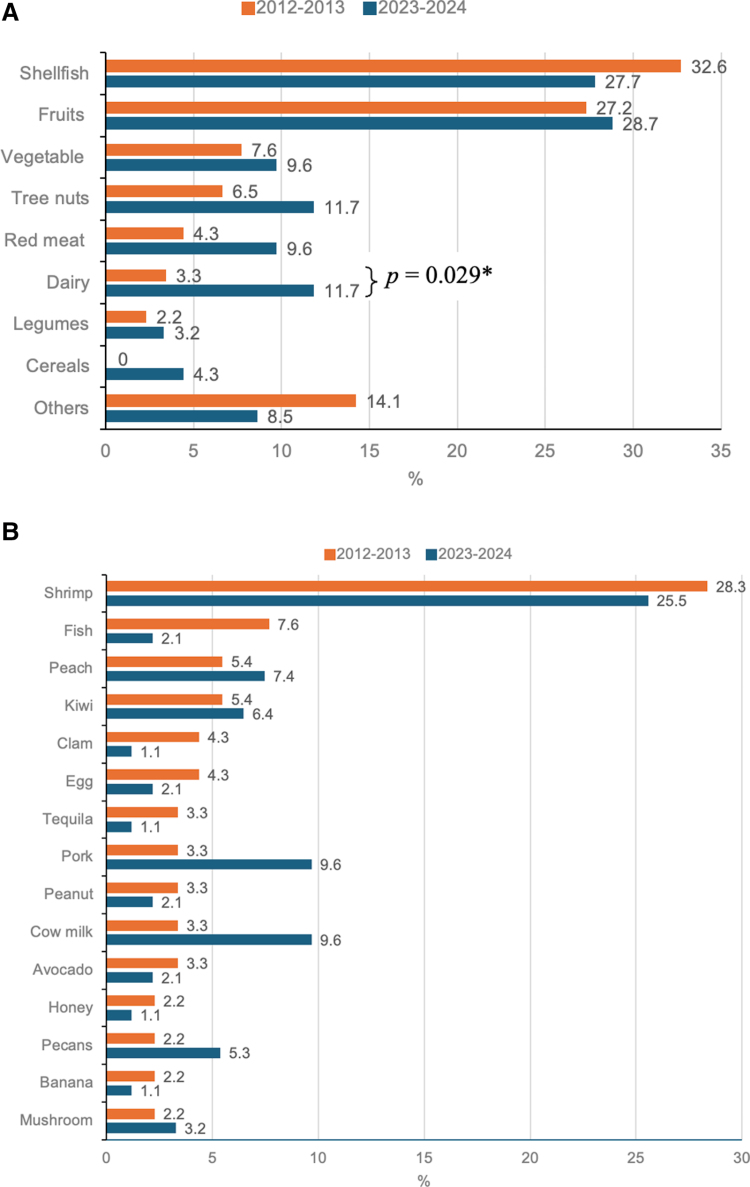
Foods most frequently associated with adverse food reactions among adults; (A) food categories and (B) individual foods. Values are expressed as percentages. Comparison of percentages using the chi-square test. Except for dairy products (*P* = 0.029), no statistically significant differences were found between the time points in the rest of the variables (2012–2013 vs 2023–2024, *P* > 0.05).

In both periods of analysis, seafood and fruits predominated as the categories most frequently associated with AFR; however, no significant differences were observed. However, a significant increase in hypersensitivity to dairy products was observed between the 2 study periods (3.3% vs 11.7%, *P* = 0.029) (Fig. 2A). When foods were analyzed individually, shrimp, fish, peach, and kiwi were the foods most frequently causing symptoms during both periods of analysis, while honey, nuts, banana, and mushrooms caused symptoms less frequently. In none of the cases was a significant difference observed between the 2 groups (*P* > 0.05) (Fig. 2B).

**Figure 3.**
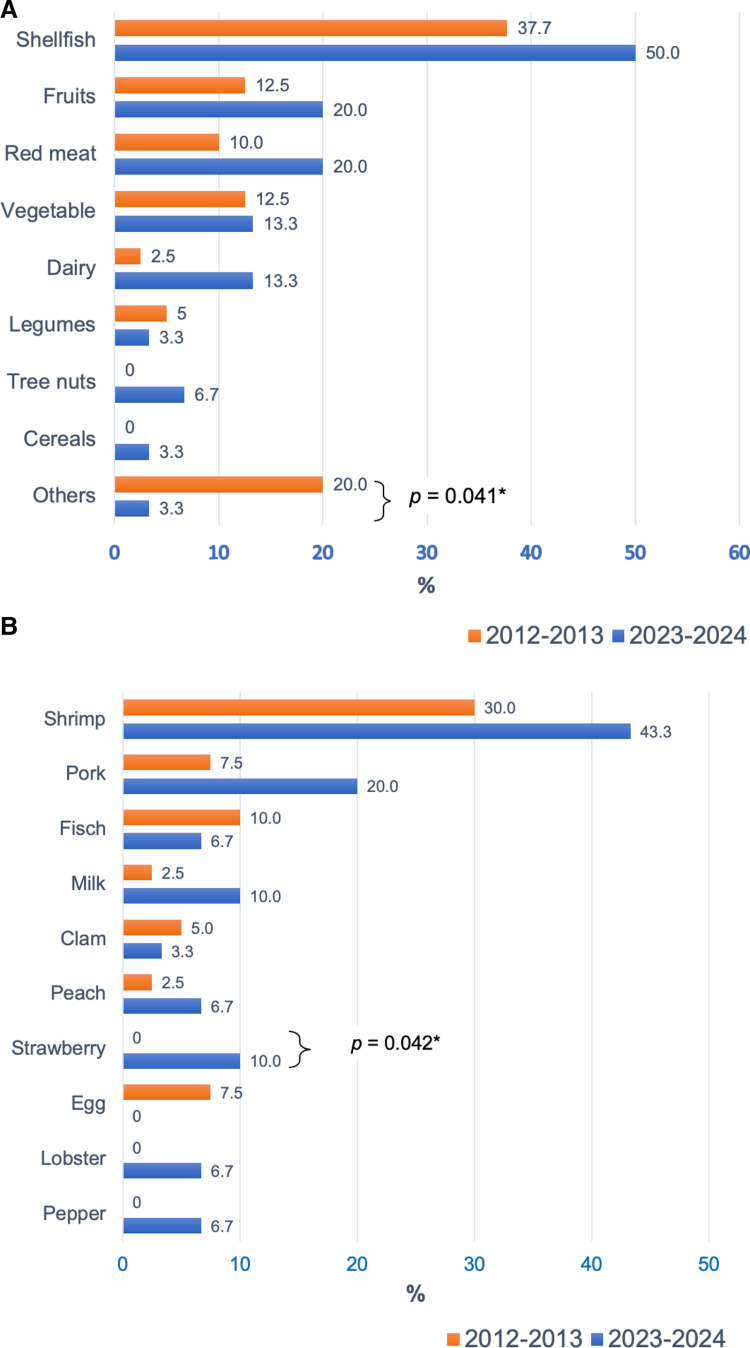
Foods most frequently associated with convincing food allergy among adults; (A) food categories and (B) individual foods. Values are expressed as percentages. Comparison of percentages using the chi-square test. Except for strawberry (*P* = 0.042), no statistically significant differences were found between the time points in the rest of the variables (2012–2013 vs 2023–2024, *P* > 0.05).

When analyzing only the group of convincing FA, once again, shellfish and fruits were the foods most associated with ailments, with no significant differences observed between the study periods (*P* > 0.05). In this case, there was a decrease in the “other foods” category (20.0% vs 3.3%, *P* = 0.041) (Fig. 3A). However, individually, shrimp and pork were the predominant foods, with no noticeable changes between the 2 study periods. Notably, strawberries were the only food that showed a significant increase (Fig. 3B).

### 3.5. Factors associated with AFR

A multivariate analysis that included 2 models conducted with all adults who participated in both study periods (cross-sectional samples for 2012 to 2013 and 2023 to 2024) showed that the factors associated with AFR were being a woman (adjusted odds ratio [OR]: 1.53, *P* = 0.013), being between 25 and 50 years of age (adjusted OR: 0.59, *P* = 0.002), having a personal background of AR (adjusted OR: 2.10, *P* = 0.004), or having AD (Adjusted OR: 4.99, *P* < 0.001) (Table 3).

**Table 3.** Factors associated with adverse food reactions among adults

	Unadjusted model			Adjusted model		
	OR	95% CI	*P*	OR	95% CI	*P*
Sex						
Male	1			1		
Female	1.52	1.09–2.12	0.014	1.53	1.09–2.13	0.013
Age, y						
18–24	1			1		
25–50	0.60	0.43–0.84	0.003	0.59	0.42–0.83	0.002
Asthma						
No	1			1		
Yes	1.32	0.71–2.46	0.377	-	-	-
Allergic rhinitis						
No	1			1		
Yes	2.00	1.20–3.34	0.008	2.10	1.28–3.46	0.004
Atopic dermatitis						
No	1			1		
Yes	4.74	2.45–9.17	<0.001	4.99	2.61–9.55	<0.001

Both models included subjects from cross-sectional 2012–2013 and 2023–2024 (n = 956). The methods of entering predictor variables were in the unadjusted model: “Enter” and in the adjusted model: “Forward: conditional.”

CI, confidence interval; OR, odds ratio.

## 4. Discussion

To our knowledge, this study is the first in Latin America to analyze the trend in the prevalence of AFR in adults and to show that it has not changed over 2 periods separated by 11 years. It also demonstrated that, with the exception of anaphylaxis, the clinical manifestations of AFR did not show notable changes; the same was observed with the prevalence of the major food categories implicated. Finally, our study identified sex, age, and personal background of AR or AD as the factors associated with AFR.

Comparing the results obtained in the 2 study periods allowed us to observe that the prevalence of AFR in adults did not change over the 11-year period. In America, the self-reported prevalence of food allergies has increased in recent years. For example, in the United States, the prevalence increased from 9.0% in 2001 to 14.9% in 2006, and finally to 13.0% in 2010 [5]; similar findings were observed in Canada, where the prevalence was 7.2% in 2010 and 9.5% in 2016 [3]. In Europe, a meta-analysis showed interesting results by differentiating between the cumulative and point prevalence of self-reported food allergies. The cumulative prevalence did not experience significant changes: 17.3% between 2000 and 2012 and 19.8% between 2012 and 2022. In contrast, the point prevalence increased from 5.9% to 19.8% during the same period of time [4]. In Asia, the prevalence of self-reported food allergies has shown a noticeable increase rising from 6.4% in 2004 to 12.5% in 2017 [6]. Other studies that have evaluated the incidence, rather than the prevalence, of food allergies have also shown equally diverse results. A couple of studies conducted in the United States showed a sustained increase in the incidence of food allergies, which began in the 2002 to 2003 biennium and tended to stabilize between 2008 and 2013, before rising again until 2018 [10, 11]. Additionally, it has also been observed that the prevalence of allergic sensitization to foods and the number of hospitalizations due to food-associated problems have increased over the years [12, 13]. In summary, our study does not support an increase in the prevalence of AFR in adults. It is likely that the existence of previous studies conducted before our first analysis (2012–2013 cross-sectional study) would have allowed us to determine whether there was an increase in the prevalence of AFR, as has been observed in some regions of the world, or if it has remained constant in our city.

Our study not only shows that the prevalence of AFR and its symptoms have remained stable over a period of 11 years but also indicates that the food groups and the foods most frequently related to its expression have remained constant, including seafood, fruits, and vegetables. What events could explain this behavior? At least 2 studies have shown that dietary habits have changed little in recent years. One of them included data from 185 countries, showing that the quality of the diet did not change significantly between 1990 and 2018, suggesting that the pattern of food consumed 30 years ago has remained constant to the present day [14]. The other study was conducted in our country; the National Health Survey clarified that the pattern of food consumption has not experienced significant changes in previous years [15]. Since the dietary pattern has not changed over time, environmental changes or urbanization over the last decade are unlikely to explain the lack of changes in the prevalence of adverse reactions and allergic diseases in adults. Why do these prevalences remain stable despite global environmental changes? The answer is beyond our research objectives, so future studies should address it.

Several risk factors have been associated with AFR; in this study, female sex and a personal history of AR or AD were factors associated with AFR in adults. On the other hand, adults aged 25 to 50 years were less likely to suffer from AFR than adults aged 18 to 24 years. In the United States, data from over 40,000 adults were analyzed, and it was found that 19% of the participants reported being allergic to food. Similar to our research, the authors observed that women and a personal history of allergic diseases were related to AFR; it was also observed that the age group of 40 to 49 had a lower risk of AFR compared with the 18 to 29 age group [1].

A significant finding was that the prevalence of self-reported anaphylaxis decreased significantly in the second period compared to the first. This result must be interpreted cautiously, given that the subjects in the first survey were not the same as those in the second. The significant difference could be due to chance, even though the subjects were randomly selected in both surveys (possible random error). On the other hand, although the prevalence of systemic symptoms did not change over time, it is important to note that in both studies, these symptoms could have corresponded to anaphylactic reactions that the subjects may not have been aware of. In any case, if the difference found between anaphylaxis prevalence was true, the causal factors of this observed decrease deserve new research since they are beyond the objectives of our study; further research is needed. Our study also showed that the prevalence of diseases such as asthma, AR, AD, urticaria, and drug allergy did not change significantly during the analyzed period, which is in line with our observation that the prevalence of AFR remained stable. In this regard, the available evidence shows a complex picture, with regions of the world experiencing increases or decreases in the prevalence of allergic diseases, whereas in others, no significant changes are observed. This is the case in Korea, where over a 10-year period, it was observed that the prevalence of asthma remained unchanged, but for AR, increases or stability in its prevalence was observed; in AD, a decrease was recorded during early childhood, but an increase during school age and in the elderly [16]. In another example, the number of cases of asthma, AR, and AD in Mexico increased from 2007 to 2010, followed by a sustained decrease until 2019 [17]. It seems that, at least for our city, the prevalence of allergic and food-related diseases has not increased in the last 11 years.

Among the limitations of this study, one notable aspect is that the data were obtained through a survey of individuals engaging in physical activities in the central streets of an industrialized city, thus not necessarily reflecting what might happen in rural regions or among people who do not frequently occur in these areas; additionally, it is possible that individuals with severe reactions associated with food consumption did not participate in physical activities, and therefore were not included in the study. Additionally, epidemiological studies based on surveys tend to overestimate the prevalence of AFR compared with studies based on clinical interviews, IgE-mediated allergic sensitization tests, or oral provocation tests. Due to the nature of the study, it was also not possible to distinguish between IgE- and non-IgE-mediated symptoms. On the other hand, the significant increase in the prevalence of dairy-related symptoms might be due to food intolerance rather than allergy; once we analyzed the adult population with convincing symptoms of food allergy, the difference was no longer significant (Fig. 3A). The age of onset of symptoms associated with food consumption was not indicated, so there is a possibility that some adults may have experienced food hypersensitivity in early stages of life and no longer have it currently. Regarding other significant limitations, the questionnaire did not include sociodemographic variables such as ethnic origin, length of residence in Guadalajara, socioeconomic status, and education level. Although the lack of these variables does not reduce the validity of the prevalences found, they would have been of great value in the search for factors associated with AFR among adults due to their possible confounding effect. In new studies like this, these variables definitely must be included. Another limitation was that the nonresponse rate was not measured; however, the probability of selection bias should have been low given that the survey was answered anonymously and sampling was probabilistic. Again, in future studies of this type, it will be relevant to measure that the proportion of subjects who decide not to participate does not exceed 20% to guarantee the validity of the results. Among the strengths of the study, consistency in methodology across both study periods stands out as well as the collection of data conducted in person by properly trained personnel, ensuring the reliability of the obtained data.

This study is the first in Latin America to demonstrate that the prevalence of AFR in Mexican adults has remained unchanged over the past 11 years. This occurrence contrasts notably with the increases observed in some regions of the world. Furthermore, except for food-associated anaphylaxis, which showed a decrease in prevalence, the rest of the symptoms also remained stable; the same can be said for the food groups most frequently implicated with AFR. It is worth highlighting that our data were collected predominantly in the pre-COVID era (2012–2024). However, it will be interesting to continue this series of cross-sections in the future to investigate whether long-COVID is related to the epidemiology of food-allergic diseases. In conclusion, more studies are necessary to confirm our results and better understand the trends of AFR in the Mexican population.

## Acknowledgements

We thank the Municipal Sports Council of Guadalajara for their support and assistance in facilitating our research activities at the Vía Recreactiva. We also extend our gratitude to all the participants who took part in our study at this venue.

## Conflicts of interest

The authors have no financial conflicts of interest.

## Author contributions

Study conception and design: Tonatiuh R. Bedolla-Pulido, Misael Aguilar-Panduro, Dante D. Hernández-Colín, María E. Nuñez-Nuñez. Acquisition of data: Tonatiuh R. Bedolla-Pulido, Misael Aguilar-Panduro. Analysis and interpretation of data: Jaime Morales-Romero, Martín Bedolla-Barajas, Dante D. Hernández-Colín, María E. Nuñez-Nuñez. Writing of the manuscript: Jaime Morales-Romero, Martín Bedolla-Barajas.

## Supplementary material

Supplementary Material 1 can be found via 10.5415/apallergy.2022.12.e38

Supplementary Material 1

Click here to view
